# Clinical significance of stromal ER and PR expression in periampullary adenocarcinoma

**DOI:** 10.1186/s40364-019-0176-9

**Published:** 2019-11-19

**Authors:** Gustav Andersson, Sebastian Lundgren, Margareta Heby, Björn Nodin, Jacob Elebro, Karin Jirström

**Affiliations:** Division of Oncology and Pathology, Department of Clinical Sciences Lund, Lund University, Lund, Sweden

**Keywords:** Periampullary adenocarcinoma, Pancreatic cancer, Estrogen receptor, Progesterone receptor, *KRAS*, Prognosis

## Abstract

**Background:**

Tamoxifen treatment has previously been reported to confer life-prolonging effects in patients with advanced pancreatic cancer, and most evidently so in women. None of these trials did however include biomarkers, and the relevance of female hormone signaling in pancreatic or other periampullary adenocarcinoma remains largely unexplored. The aim of this study was to examine the extent and potential clinical significance of estrogen receptor-α (ER) and progesterone receptor (PR) expression in pancreatic and other periampullary cancers.

**Methods:**

ER and PR expression was examined using immunohistochemistry on tissue microarrays with primary tumors from a retrospective consecutive cohort of 175 patients with resected periampullary adenocarcinoma, with long-term clinical follow-up. Non-parametric and Chi square tests were applied to examine the associations of stromal ER and PR expression with patient and tumor characteristics. Kaplan-Meier analysis and log rank test were applied to illustrate survival differences in relation to ER and PR expression. Cox regression proportional hazards models were applied to examine the associations between investigative factors and risk of death and recurrence, and to test for interactions between *KRAS* mutation status and hormone receptor expression in relation to survival.

**Results:**

Expression of both ER and PR was more frequent in the tumor-associated stroma than in the epithelium. A significant prognostic interaction, independent of tumor morphology, was found between stromal PR expression and *KRAS* mutation status in relation to both overall and recurrence-free survival (p_interaction_ = 0.026 and p_interaction_ = 0.005), in particular in women (p_interaction_ = 0.002 and p_interaction_ = 0.005). Specifically, stromal PR expression was associated with a prolonged survival in patients with *KRAS*-mutated tumors, whereas the opposite was seen for *KRAS* wild-type tumors. The prognostic value of ER positivity was limited to the subgroup of women with tumors of pancreatic origin.

**Conclusions:**

These results demonstrate that stromal PR rather than ER expression, together with *KRAS* mutation status, provides long-term prognostic information in patients with periampullary adenocarcinoma. Further study into the mechanistic basis for these observations may unveil important clues to the pathogenesis of these cancers and open up for the discovery of novel treatment options.

## Background

Pancreatic cancer is an almost uniformly fatal disease, from which most patients decease within 1 year, and thus, the incidence approximates the prevalence [1]. It is the most common tumor within the clinical entity of periampullary tumors, including tumors originating in the distal bile duct, pancreas, ampulla of Vater and the periampullary duodenum. Information on morphological type, i.e. intestinal (I-type) or pancreatobiliary type (PB-type), has been shown to provide prognostic information, with the latter having the poorest outcome [2–5]. Only 15–20% of the tumors are resectable at presentation [6]. Of the remaining majority, 30–40% present with locally advanced tumor growth, leaving almost 40–55% of all patients with stage IV disease [7]. Recent and growing data support the use of neoadjuvant chemotherapy, and for patients presenting with a borderline resectable tumor, approximately one-third can be converted to resectability resulting in a doubled overall survival compared to those who remain unresectable [8]. Adjuvant chemotherapy is the standard of care following resection for pancreatic adenocarcinoma. In the palliative setting, survival is strictly limited, and only modestly improved by chemotherapy [9, 10]. Although the vast majority of pancreatic cancers contain somatic mutations [11], there is still a complete lack of “actionable” molecular targets, and immunotherapies have not proven to be successful [12]. Accordingly, there is an urgent need for novel treatment alternatives and suitable biomarkers to improve the outlook for these patients. In this context, it is noteworthy that several trials in the 1980s to 1990s reported beneficial effects of tamoxifen treatment in patients with pancreatic adenocarcinoma [13–15], primarily in women with advanced disease [16, 17]. None of these studies did however include biomarker analyses, nor did they consider the full range of periampullary cancers.

Whilst the role of female hormone signaling in established pancreatic adenocarcinomas remains unclear, several studies, including research from our group, have suggested an inverse relationship between exogenous and endogenous female sex hormones and risk of pancreatic cancer [18–26]. Contrasting findings have however been presented by others [23, 25, 27–31].

Evidently, the interplay between hormonal factors and pancreatic cancer is complex and far from being fully understood. Only a few previous studies have examined the expression of estrogen receptor-α (ER) and progesterone receptor (PR) in pancreatic or other periampullary adenocarcinomas [32, 33], and only the latter assessed their prognostic value. The aim of the present study was therefore to investigate the expression of ER and PR in tumors from a well-characterized, consecutive retrospective cohort of resected periampullary adenocarcinoma, with particular emphasis on their relationship with the mutational landscape, clinicopathological characteristics and long-term survival.

## Methods

### Study cohort

The study cohort, previously described in more detail [34–38], is a retrospective consecutive cohort encompassing 175 patients diagnosed with pancreatic or other periampullary adenocarcinomas, 65 cases with I-type morphology and 110 cases with PB-type morphology. All tumors were surgically resected through the Whipple procedure (pancreaticoduodenectomy) between Jan 1st 2001 and Dec 31st 2011, in Malmö and Lund University Hospitals, Sweden. Hematoxylin and eosin stained tumor slides from all cases were re-evaluated by a board-certified pathologist (JEL), blinded to original reports and patient outcomes. Evaluation of tumor origin and morphology was based on previously described criteria [34].

Baseline was set at the date of surgery, the primary endpoint was overall survival (OS) and the secondary endpoint recurrence-free survival (RFS), set at date of death and date of recurrence respectively, or March 31st 2017, the last date of follow-up, whichever came first. The Swedish National Civil Register provided data on survival, and patient records were reviewed for information on neoadjuvant and adjuvant treatment, and tumor recurrence. At last follow-up, 137 patients were dead, and 124 had denoted recurrence. The median follow-up time for the entire cohort was 29.7 (range 1.9–185.1) months, and 86.1 (range 63.5–185.1) months for patients alive [39].

### Tissue microarray construction

Tissue microarrays (TMAs) were constructed using a semi-automated arraying device (TMArrayer, Pathology Devices, Westminster, MD, USA). A standard set of three tissue cores (1 mm diameter) were obtained from each of the 175 formalin-fixed paraffin-embedded (FFPE) primary tumors, whenever possible from different donor blocks, and one to three tissue cores from lymph node metastases of 105 cases. Additionally, non-malignant pancreatic tissue samples were obtained from 50 of the resection specimens, using a standard set of two tissue cores (1 mm diameter).

### Immunohistochemistry and staining evaluation

For immunohistochemical analysis, 4 μm TMA-sections were automatically pre-treated using ULTRA Cell Condition Solution 1, pH 8.5 (Ventana Medical Systems, Tucson, AZ, USA) for heat induced epitope retrieval, and stained with the monoclonal antibodies CONFIRM anti-ER (SP1) and anti-PR (1E2) in a Ventana BenchMark stainer (Ventana Medical Systems). The antibody-antigen complex was visualized with UltraView Universal diaminobenzidine (DAB) Detection kit (Ventana Medical Systems).

Stained slides were digitalized at 20x magnification using the automated scanning system Hamamatsu, NanoZoomer, 13239-01, Hamamatsu Photonics, Sunayama-cho, Naka-ku, Hamamatsu City, Shizuoka, Japan). The immunohistochemical staining was manually annotated by three independent observers (GA, KJ and JEL) who were blinded to the clinical data and outcome of the patients, and with the two latter being board-certified pathologists, using the NDP.view2 viewer software version 3.2.12 (Hamamatsu Photonics). Expression of ER and PR was annotated as the number of tumor-associated stromal cells with nuclear staining. Conflicting annotations were jointly re-evaluated in order to reach consensus. The intensity of staining was not evaluated.

### Next generation sequencing

DNA extraction was performed on tissue cores (1 mm diameter), obtained alongside the TMA cores from tumor cell-enriched FFPE tissue, with the Qiagen GeneRead (Qiagen, Hilden, Germany) kit for FFPE tissue in accordance with instructions from the manufacturer. For analysis, a sufficient quantity of tumor cells could be obtained from 102 (58.3%) cases, 40 (39.2%) I-type tumors and 62 (60.8%) PB-type tumors, including 10 duodenal, 42 ampullary, 30 distal bile duct and 20 pancreatic tumors. A gene panel with selected cancer-associated genes (*n* = 70) (see Additional file 1: Data 1) was assembled and characterized using Illumina TruSeq custom amplicon assay (Illumina Inc., San Diego, USA) with a MiSeq instrument according to the manufacturer’s instructions, previously described in more detail by Lundgren et al. [40]. Only exon parts of the analyzed genes were sequenced. The supplier’s standard analysis pipeline (Illumina Inc., San Diego, CA, USA) was applied for alignment, quality filtering, variant calling, and variant annotation. Only nonsynonymous variants with variant frequency ≥ 4% were included. In order to exclude SNPs frequently reported in various populations, the identified mutations were screened against the COSMIC and ExAC databases.

### Statistical analysis

In the statistical analyses, ER and PR expression were defined as continuous variables as well as binary variables, dichotomized into positive and negative, i.e. presence vs absence of positive stromal cells. Spearman’s rank correlation test was used to examine the intercorrelation between the numbers of ER positive (ER+) and PR positive (PR+) stromal cells allover, and stratified for sex and morphology. Non-parametric and Chi square tests were applied for analysis of the associations of stromal ER and PR expression with patient and tumor characteristics in the entire cohort, as well as in strata according to sex and morphology. The distribution of mutations in relation ER and PR expression, stratified by sex and morphology, was visualized by heat maps created in OncoPrinter, provided by cBioPortal Web (http://www.cbioportal.org/oncoprinter.jsp, access date: 2018-11-05) [41, 42]. Kaplan-Meier analysis and log rank test were applied to illustrate differences in OS and RFS stratified by sex, morphology, and *KRAS* mutation status in relation to ER and PR expression. Univariable and multivariable Cox regression proportional hazards modeling was applied to estimate hazard ratios (HR) and 95% confidence intervals (CI) for risk of death and recurrence. The multivariable analysis included adjustment for age at baseline (continuous), tumor morphology (I-type/PB-type), T-stage (pT1–2/pT3–4), N-stage (pN0/pN1), tumor grade (well-moderate differentiation/poor differentiation-undifferentiated), adjuvant chemotherapy treatment (no/yes), lymph vessel invasion (no/yes), blood vessel invasion (no/yes) and perineural invasion (no/yes). Cox regression proportional hazards modeling was also used to test for potential interactions between *KRAS* mutation status and biomarker (ER/PR) expression in relation to survival, using the following interaction variable: *KRAS* mutation (0/1) x biomarker (0/1).

All statistical tests were two sided and *p*-values < 0.05 were considered significant. As this study is considered exploratory, the presented nominal *p*-values were not adjusted for multiple testing [43]. All statistical analyses were computed using IBM SPSS Statistics version 25.0 (SPSS Inc., Chicago, IL, USA).

## Results

### Distribution of ER and PR expression, overall and stratified for sex and morphology

ER and PR expression was mainly detected in the peritumoral stroma. Weak epithelial expression of ER and PR was denoted in 2.9% of the cases and therefore not accounted for in the statistical analyses. Neuroendocrine islets frequently displayed moderate to strong nuclear expression of ER and PR.

**Fig. 1:**
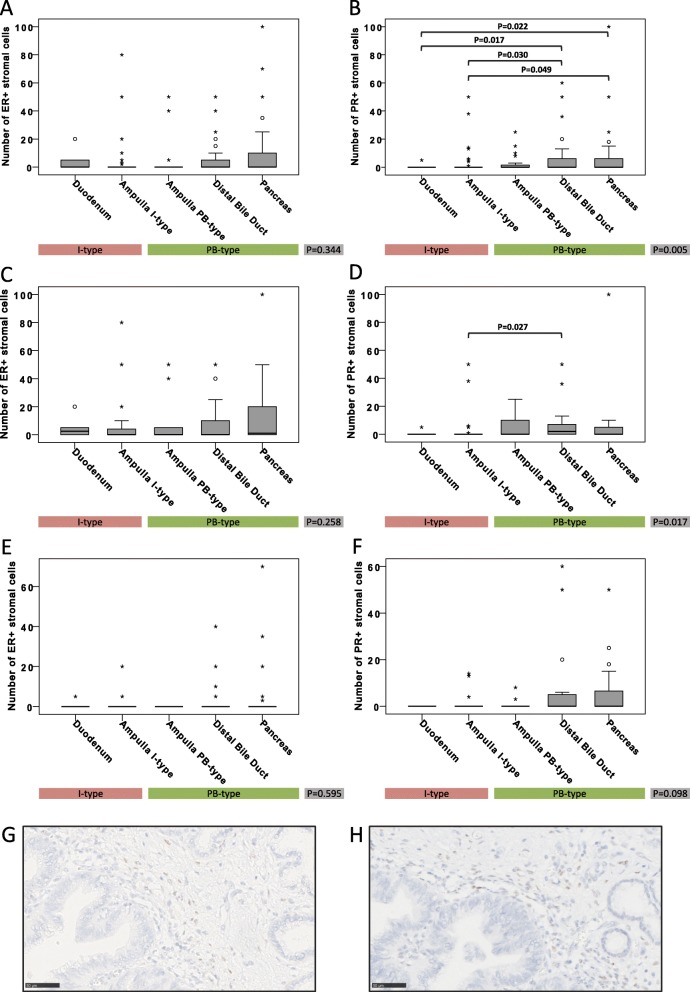
Distribution of hormone receptor positive stromal cells in relation to tumor origin and morphology. Box plots illustrating the distribution of stromal cells expressing ER (**a**, **c**, **e**) and PR (**b**, **d**, **f**) among the five different tumor origins, in the entire cohort (**a**-**b**), in women (**c**-**d**) and in men (**e**-**f**). *P*-values were measured with non-parametric test. Sample immunohistochemical images of stromal ER and PR expression, respectively, are shown in panels (**g** and **h**)

The distribution of ER and PR expression among the five different tumor origins is shown in Fig. 1, along with sample immunohistochemical images of stromal ER and PR expressing cells. Stromal ER positivity, allover denoted in 29.0% of the cases, with numbers ranging from 2 to 100 positive cells, was equally distributed between I-type and PB-type tumors (31.1% vs 25.4%, *p* = 0.43), but was more common in women compared to men (entire cohort: 41.7% vs 16.5%, *p* < 0.001 and PB-type tumors: 46.0% vs 17.9%, *p* = 0.002). However, the number of ER+ stromal cells was not associated with morphology, neither in the entire cohort nor in subgroup analysis according to sex. Stromal PR positivity, allover observed in 30.7% of the cases, with numbers ranging from 1 to 100 positive cells, was significantly more common in PB-type than in I-type tumors overall (38.1% vs 17.2% positivity, *p* = 0.006), and in women (45.8% vs 18.8% positivity, *p* = 0.013). Similarly, the number of PR+ stromal cells was significantly higher in PB-type than in I-type tumors overall (*p* = 0.005), and in women (*p* = 0.017).

As shown in Additional file 2: Table S1, there was a significant intercorrelation between stromal ER+ and PR+ in the entire cohort (*p* < 0.001), and in subgroup analysis according to sex (women: *p* = 0.011, men: *p* < 0.001). In subgroup analysis according to tumor morphology, there was a significant intercorrelation between ER+ and PR+ in PB-type tumors in the entire cohort (*p* < 0.001) and in subgroup analysis according to sex (women: *p* = 0.036, men: *p* < 0.001). There was no significant intercorrelation between ER+ and PR+ in I-type tumors.

### Associations of ER and PR with patient and tumor characteristics

**Fig. 2:**
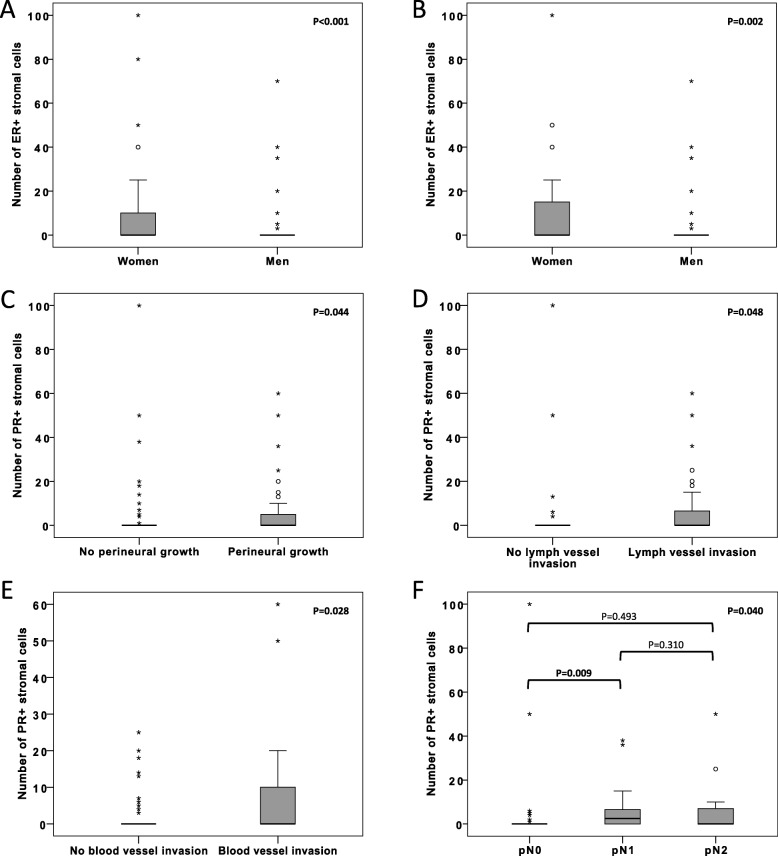
Distribution of hormone receptor positive stromal cells in relation to patient and tumor characteristics. Box plots illustrating the distribution of **a** ER expression in relation to sex in the entire cohort, **b** ER expression in relation to sex in PB-type tumors, **c** PR expression in relation to perineural growth in the entire cohort, **d** PR expression in relation to lymph vessel invasion in PB-type tumors, **e** PR expression in relation to blood vessel invasion in men, and **f** PR expression in relation to N-stage in women. *P*-values were measured with non-parametric test

Associations between binary variables of positive and negative ER and PR expression with clinicopathological characteristics are shown in Additional file 3: Table S2 and Additional file 4: Table S3, respectively. All significant associations of the number of ER and PR positive stromal cells with patient and tumor characteristics in the entire cohort, and stratified for morphology and sex, respectively, are shown in Fig. 2. There was a significant positive association between the number of ER+ stromal cells and female sex in the entire cohort and in PB-type tumors. Significant positive associations were also seen between the number of PR+ stromal cells and perineural growth in the entire cohort, lymph vessel invasion in PB-type tumors, blood vessel invasion in men, and N-stage in women.

### Distribution of common mutations in relation to ER and PR expression

**Fig. 3:**
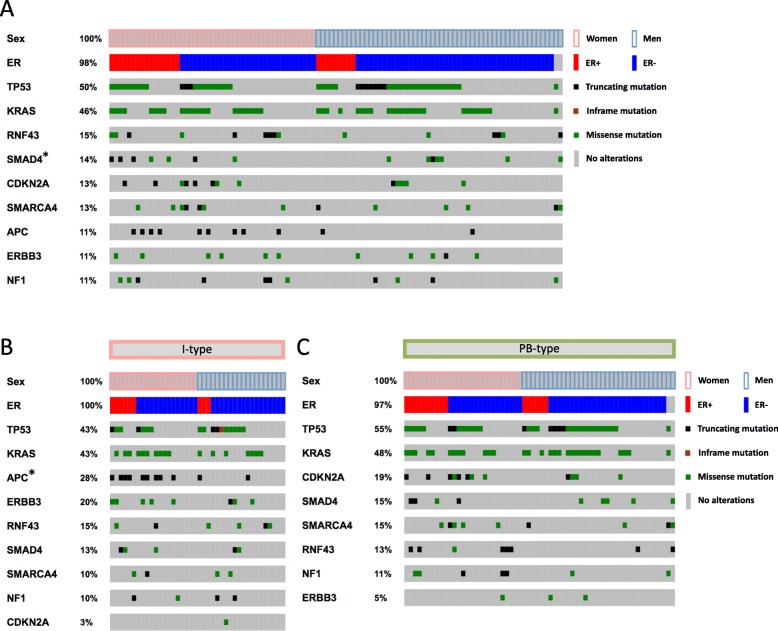
Frequency and type of mutations in relation to ER expression. Heatmaps illustrating the most common mutations, their type, and frequency in relation to ER status in **a** the entire cohort, **b** I-type tumors and **c** PB-type tumors. The genetic alterations were classified as truncating, missense or in-frame mutations. *P*-values were measured with Pearson chi-square test, and significant associations are indicated with an asterisk

**Fig. 4:**
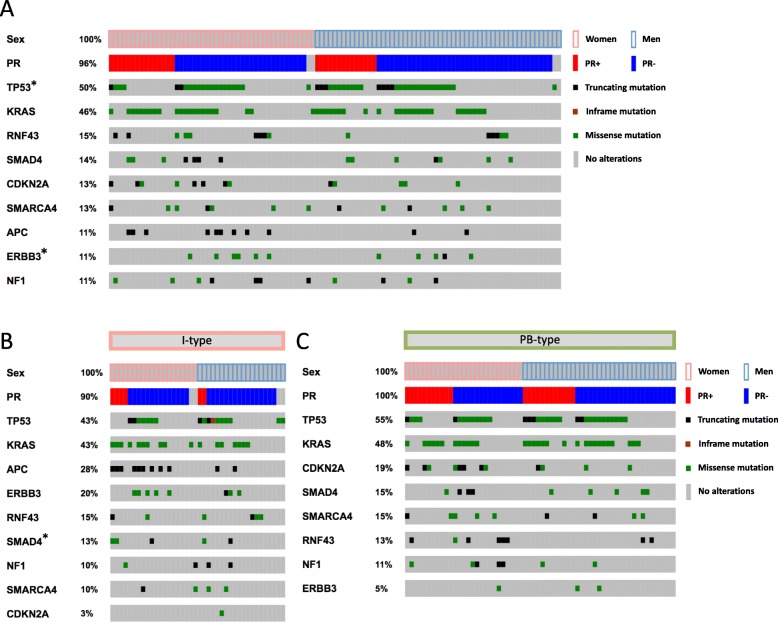
Frequency and type of mutations in relation to PR expression. Heatmaps illustrating the most common mutations, their type, and frequency in relation to PR status in **a** the entire cohort, **b** I-type tumors and **c** PB-type tumors. The genetic alterations were classified as truncating, missense or in-frame mutations. *P*-values were measured with Pearson chi-square test, and significant associations are indicated with an asterisk

Heatmaps of the distribution and type of the nine most common mutations (mutation frequency > 10%) stratified by ER and PR status and sex, in the entire cohort and further stratified by morphology, are shown in Figs. 3 and 4, respectively. The associations between binary tumor mutation status and ER and PR expression, respectively, allover and stratified for sex and morphology, are shown in Additional file 5: Table S4 and Additional file 6: Table S5. ER positivity was significantly associated with *SMAD4* mutations in women (*p* = 0.024), and with *APC* mutations, exclusively found in I-type tumors [40] (*p* = 0.032). PR positivity was significantly associated with *TP53* mutations in men (*p* = 0.044), with *SMAD4* mutations in I-type tumors (*p* = 0.005 for all, *p* = 0.043 for women), and with *ERBB3* wild-type tumors in the entire cohort (*p* = 0.021).

### Associations of stromal ER and PR expression with overall and recurrence-free survival

As shown in Table 1, neither ER nor PR expression in the tumor stroma was prognostic per se in the entire cohort, nor in subgroup analysis according to sex. There was however a significant interaction between PR, but not ER, expression and *KRAS* mutation status in relation to OS in the entire cohort (p for interaction = 0.026) and in women (p for interaction = 0.002).

**Table 1:** Unadjusted hazard ratios for death in relation to stromal ER and PR expression, allover and stratified by *KRAS* mutation status and sex, respectively

	All		*KRAS* wild-type		*KRAS* mutated		
	n (events)	HR (95% CI)	n (events)	HR (95% CI)	n (events)	HR (95% CI)	P_interaction_
ER status							
All							
ER-	118 (93)	1.00	40 (27)	1.00	34 (28)	1.00	0.694
ER+	48 (39)	0.97 (0.67–1.41)	13 (10)	1.14 (0.55–2.37)	12 (11)	0.93 (0.46–1.88)	0.757^a^
*p*		0.865		0.721		0.841	
Women							
ER-	49 (35)	1.00	17 (10)	1.00	14 (10)	1.00	0.874
ER+	35 (26)	1.05 (0.63–1.74)	8 (5)	1.09 (0.37–3.22)	8 (7)	1.35 (0.50–3.65)	0.947^a^
*p*		0.864		0.870		0.548	
Men							
ER-	69 (58)	1.00	23 (17)	1.00	20 (18)	1.00	0.387
ER+	13 (13)	1.12 (0.61–2.05)	5 (5)	1.30 (0.47–3.59)	4 (4)	0.63 (0.21–1.92)	0.516^a^
*p*		0.723		0.613		0.420	
PR status							
All							
PR-	111 (86)	1.00	38 (24)	1.00	29 (26)	1.00	0.026
PR+	49 (43)	1.25 (0.87–1.81)	12 (11)	1.94 (0.94–3.98)	17 (14)	0.66 (0.34–1.27)	0.037^a^
*p*		0.234		0.071		0.216	
Women							
PR-	52 (36)	1.00	18 (9)	1.00	12 (11)	1.00	0.002
PR+	28 (24)	1.35 (0.80–2.27)	6 (6)	2.85 (1.01–8.09)	9 (6)	0.030 (0.11–0.85)	0.004^a^
*p*		0.257		0.048		0.024^b^	
Men							
PR-	59 (50)	1.00	20 (15)	1.00	17 (15)	1.00	0.678
PR+	21 (19)	1.23 (0.72–2.09)	6 (5)	1.32 (0.47–3.70)	8 (8)	1.70 (0.71–4.07)	0.785^a^
*p*		0.452		0.592		0.234	

^a^Interaction analysis including adjustment for morphological type (I-type/PB-type)

^b^Hazard ratio adjusted for morphological type 0.28 (0.10–0.80) in women

Similar findings were seen for PR expression and *KRAS* mutation status in relation to RFS in the entire cohort (p for interaction = 0.005) and in women (p for interaction = 0.005), as demonstrated in Table 2. Of note, there was also a significant interaction between ER expression and *KRAS* mutation status in relation to RFS in men (p for interaction = 0.025). However, the impact of ER expression on RFS did not reach significance in any strata according to *KRAS* mutation status.

**Table 2:** Unadjusted hazard ratios for recurrence in relation to stromal ER and PR expression, allover and stratified by *KRAS* mutation status and sex, respectively

	All		*KRAS* wild-type		*KRAS* mutated		
	n (events)	HR (95% CI)	n (events)	HR (95% CI)	n (events)	HR (95% CI)	P_interaction_
ER status							
All							
ER-	118 (84)	1.00	40 (23)	1.00	34 (29)	1.00	0.078
ER+	48 (36)	0.90 (0.61–1.33)	13 (10)	1.49 (0.71–3.15)	12 (8)	0.54 (0.24–1.19)	0.056^a^
*p*		0.587		0.294		0.127	
Women							
ER-	49 (32)	1.00	17 (9)	1.00	14 (10)	1.00	0.544
ER+	35 (24)	0.97 (0.57–1.65)	8 (5)	1.33 (0.44–4.00)	8 (5)	0.84 (0.28–2.53)	0.425^a^
*p*		0.905		0.615		0.760	
Men							
ER-	69 (52)	1.00	23 (14)	1.00	20 (19)	1.00	0.025
ER+	13 (12)	1.02 (0.54–1.92)	5 (5)	2.30 (0.78–6.78)	4 (3)	0.31 (0.08–1.11)	0.015^a^
*p*		0.951		0.131		0.072	
PR status							
All							
PR-	111 (77)	1.00	38 (21)	1.00	29 (26)	1.00	0.005
PR+	49 (40)	1.25 (0.85–1.83)	12 (10)	2.07 (0.97–4.42)	17 (12)	0.50 (0.25–0.99)	0.008^a^
*p*		0.252		0.061		0.048^b^	
Women							
PR-	52 (33)	1.00	18 (9)	1.00	12 (10)	1.00	0.005
PR+	28 (22)	1.35 (0.79–2.33)	6 (5)	2.71 (0.89–8.26)	9 (5)	0.32 (0.11–0.97)	0.011^a^
*p*		0.276		0.080		0.043^b^	
Men							
PR-	59 (44)	1.00	20 (12)	1.00	17 (16)	1.00	0.337
PR+	21 (18)	1.20 (0.69–2.08)	6 (5)	1.64 (0.58–4.70)	8 (7)	0.84 (0.34–2.09)	0.249^a^
*p*		0.510		0.353		0.715	

^a^Interaction analysis including adjustment for morphological type (I-type/PB-type)

^b^Hazard ratio adjusted for morphological type 0.46 (0.23–0.93) in the entire cohort, and 0.32 (0.11–0.96) in women

As further shown in Tables 1 and 2, the interactions between PR expression and *KRAS* status in relation to both OS and RFS remained significant after adjustment for morphological type.

The independent prognostic impact of ER and PR expression, respectively, in the entire cohort was also tested in a multivariable model including other established prognostic factors, whereby no significant associations were found (data not shown). Due to the small number of cases, adjustment for other factors than morphological type was not performed in further substrata.

Subgroup analysis according to morphological type did not reveal any significant associations between ER or PR expression with neither OS nor RFS (data not shown). However, subgroup analysis according to anatomical tumor origin revealed that ER positivity was significantly associated with a prolonged OS and RFS in women with tumors of pancreatic origin in univariable analysis (HR 0.18, 95% CI 0.05–0.60 and HR 0.18, 95% CI 0.05–0.68, respectively), but not in multivariable analysis. PR+ was not prognostic in subgroup analysis according to tumor origin (data not shown).

**Fig. 5:**
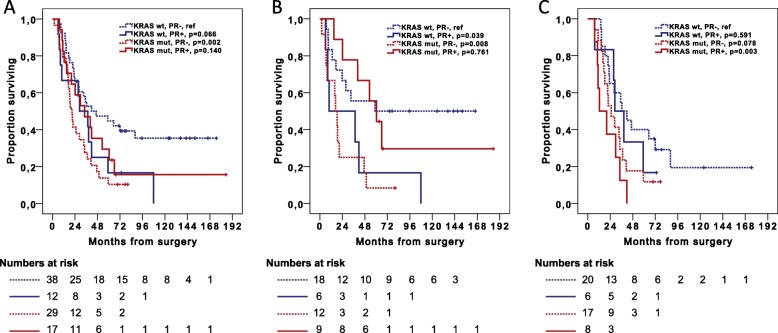
Associations of *KRAS* mutation status and PR-expression with overall survival. Kaplan-Meier curves illustrating overall survival in relation to *KRAS* mutation status and PR status in **a** all cases, **b** women and **c** men. *P*-values were measured with log-rank test

**Fig. 6:**
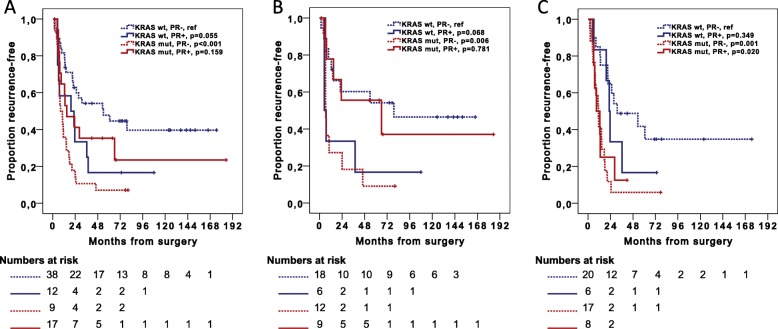
Associations of *KRAS* mutation status and PR-expression with recurrence-free survival. Kaplan-Meier curves illustrating recurrence-free survival in relation to *KRAS* mutation status and PR status in **a** all cases, **b** women and **c** men. *P*-values were measured with log-rank test

Kaplan-Meier analyses of OS and RFS in combined strata of PR expression and *KRAS* mutation status are shown in Figs. 5 and 6, respectively. In the entire cohort, median OS was 34.3 months for patients with PR+/*KRAS*-mutated tumors vs 20.9 months for patients with PR−/*KRAS*-mutated tumors, and 28.8 months for patients with PR+/*KRAS* wild-type tumors vs 41.8 months for patients with PR−/*KRAS* wild-type tumors. In women, median OS was 60.5 months for patients with PR+/*KRAS-*mutated tumors vs 16.6 months for patients with PR−/*KRAS-*mutated tumors, and 9.9 months for patients with PR+/*KRAS* wild-type tumors vs 59.0 months for patients with PR−/*KRAS* wild-type tumors.

In the entire cohort, median RFS was 15.0 months for patients with PR+/*KRAS-*mutated tumors vs 8.2 months for patients with PR−/*KRAS-*mutated tumors, and 19.3 months for patients with PR+/*KRAS* wild-type tumors vs 53.9 months for patients with PR−/*KRAS* wild-type tumors. In women, median RFS was 66.0 months for patients with PR+/*KRAS-*mutated tumors vs 6.7 months for patients with PR−/*KRAS-*mutated tumors, and 5.1 months for patients with PR+/*KRAS* wild-type tumors vs 79.2 months for patients with PR−/*KRAS-*wild-type tumors. Neither ER nor PR expression was prognostic in relation to mutation status of any other of the most frequently mutated genes (data not shown).

## Discussion

This study provides a first description of the prognostic significance of stromal ER and PR expression in pancreatic and other periampullary cancers. Both receptors were expressed mainly in the stromal compartment, in approximately one third of cases but not entirely overlapping. Whilst ER expression was only prognostic in the subgroup of women with pancreatic cancer in unadjusted analysis, stromal PR positivity conferred a contrasting prognostic value dependent on *KRAS* mutation status in the entire cohort, and in particular in women. Specifically, *KRAS* mutated/PR positive tumors signified a prolonged survival, whereas *KRAS* wild-type/PR positive tumors signified a reduced survival, and these associations were reflected in both OS and RFS.

The stromal component of ER and PR adjacent to the invasive tumors in this study bears some resemblance to the ovarian-type stroma (OTS) that characterizes pancreatic mucinous cystic neoplasms (MCN), potential premalignant lesions mainly occurring in the tail of the pancreas, and in particular in middle-aged women. The presence of an OTS, together with a lack of communication with the main pancreatic duct, distinguishes these entities from intraductal papillary mucinous neoplasms, another type of premalignant lesion [44, 45]. The nature of this OTS is debated; while some authors suggest that it constitutes an embryologic remnant of the ovary [46], some speculate that it derives from fetal remnants of periductal mesenchyme exposed to the hormonal environment in perimenopausal women, and that the OTS in turn stimulates the adjacent epithelium to proliferate and give rise to MCN [47]. Loss of PR expression in MCN has been demonstrated to correlate with high-grade dysplasia/carcinoma in-situ or true invasion [48]. Conversely, the frequency of *KRAS* mutations in MCN increases with the grade of dysplasia and/or invasion [49]. In the present study, no significant associations were found between stromal ER or PR expression and *KRAS* mutation status, neither overall nor in any subgroup analyses. Moreover, as previously shown, the frequency of *KRAS* mutations does not differ significantly between I-type and PB-type tumors [40]. The observation of a prognostic interaction of PR expression and *KRAS* mutation status is however noteworthy, as it suggests that PR-mediated signaling may have tumor suppressing effects in *KRAS*-mutated tumors and tumor-promoting effects in *KRAS* wild-type tumors. Speculatively, these effects may be more prominent in the hormonal milieu of the female pancreas, but it must be pointed out that while the findings in this study indicate a potential effect of sex on the prognostic significance of PR/*KRAS* mutation status, there was no significant interaction with sex in relation to clinical outcome.

The specific functions of steroid hormone receptors are known to be dependent on the tissue-specific as well as cell-specific context, and, as previously mentioned, the mechanistic basis for their potential action in pancreatic carcinogenesis remains to be explored. Some clues may however be derived from studies on other types of cancer. For instance, the importance of stromal PR in endometrial cancer has been highlighted in a paper by Janzen et al. [50], wherein the authors demonstrate that the effects of progesterone are mediated via paracrine signaling via PR in the tumor microenvironment. Of particular interest are the findings that concomitant *KRAS*-activation and *PTEN*-loss induced progesterone-resistance and loss of stromal PR in endometrial tumors, but that progesterone sensitivity could be reintroduced through exogenous administration of PR into the stroma. The authors conclude that analysis of *PTEN* and *KRAS* status in the epithelium of the tumor, and PR expression in the stroma, may be suitable biomarkers for progesterone sensitivity in endometrial cancer [50]. With these data in mind, the finding of a prognostic interaction between *KRAS* and PR in the present study suggest that further in-depth interrogation into the functional link between *KRAS* and stromal PR-signaling in pancreatic and other periampullary cancers may unveil important clues to their pathogenesis and open up for the development of novel treatment strategies.

Herein, well-validated antibodies routinely used in the clinic were applied to detect ER and PR expression, and the expression in tumor cells was found to be negligible. One recent study reported both ER and PR to be weakly expressed in a proportion of 60 pancreatic ductal adenocarcinoma cases [33]. In that study, expression of both ER-α and ER-β, as well as PR and androgen receptors, was analyzed by an automated approach resulting in an H-score, and while the exact proportion of cases with positive vs negative expression was not denoted, only ER-α expression was found to be a negative prognostic factor, however not in adjusted analysis [33]. Results from two other recent studies suggest that expression of ER-β [51], as well as phosphorylated ER-β [52], are negative prognostic factors in pancreatic ductal adenocarcinoma. The issue with a lack of reliable antibodies for detection of ER-β has however not yet been resolved [53], and therefore these findings should be interpreted with caution.

A potential limitation to the present study is the use of the TMA technique, which may not accurately reflect the spatial heterogeneity of investigative biomarkers. It must however be pointed out that not even analysis of a whole tissue section from one donor block would help circumvent this limitation. In the TMA used in the present study, each tumor is, with few exceptions, represented by tissue cores derived from different donor blocks.

Another potential limitation is the heterogeneity within periampullary adenocarcinoma. From a clinical viewpoint however, it is still relevant to consider these tumors as one entity, as anatomic origin cannot always be determined for irresectable tumors, which constitute a vast majority of the cases.

Given the scattered distribution of hormone receptor positive cells from neuroendocrine islets in resected tumor specimens, assessment of ER or PR mRNA levels is not likely to provide reliable prognostic information, but may give some directions. For instance, assessment of ER-α, ER-β and PR levels in 176 pancreatic ductal adenocarcinoma cases in The Cancer Genome Atlas (TCGA), as displayed in the Human Protein Atlas/Pathology Atlas portal (www.proteinatlas.org), reveals an overall low mRNA expression of ER-α, ER-β and PR (average FPKM 0.1, 0.2 and 0.4, respectively). Moreover, high levels of PR and ER-β are shown to be weakly associated with an improved survival, whereas ER-α is not prognostic. Of note, > 90% of the TCGA pancreatic cancers are *KRAS*-mutated. Thus, although the assay is not optimal, the findings regarding PR mRNA expression in the TCGA can be interpreted as being supportive of the herein presented results.

Regarding the associations of other common mutations with stromal ER or PR expression, no clear patterns or associations were found in the present study, and since many subgroups were analyzed, the few significant associations that were found may well be due to type 1 errors. Moreover, although the herein analyzed consecutive cohort of resected periampullary adenocarcinoma is a clinically and histopathologically well-characterized consecutive series of all patients who underwent pancreaticoduodenectomy during an 11-year period, mutation analyses could only be performed in 102 cases from the original cohort of 175 cases, and further stratifying for sex or morphology renders rather small subgroups available for statistical analyses. However, since the outlook for patients afflicted by pancreatic or other periampullary cancers is sinister and treatment options are limited, identification of novel therapies with clinical benefit only in small subgroups would still be a great leap forward.

Although not reporting other than the relationship between stromal ER and PR expression and clinical outcome upon standard treatment in a retrospective, consecutive cohort, the results from this study encourage a revision of some earlier studies that demonstrated beneficial effects of tamoxifen treatment in patients with advanced pancreatic adenocarcinoma [15], in particular in women [16, 17]. None of these clinical trials included any biomarkers, and the anti-cancer effects of tamoxifen in pancreatic cancer may well be exerted via other mechanisms than blocking of ER-mediated signaling. For instance, tamoxifen has also been shown to confer anti-angiogenic effects [54], partly through inhibition of protein kinase C [55, 56], and partly by diminishing the release of vascular endothelial growth factor from thrombocytes [57], and anti-metastatic effects, through prevention of platelet-assisted migration of tumor cells through the endothelium [57]. Nevertheless, a role of female hormone receptors in this context cannot be ruled out.

## Conclusion

The results from this study demonstrate that stromal PR expression, together with *KRAS* mutation status, provides long-term prognostic information in particular in female patients with pancreatic and other periampullary cancers. Further study into the functional link between *KRAS* and stromal PR-mediated signaling in these cancers is encouraged, as this may unveil important clues to their pathogenesis and open up for the discovery of novel treatment options. The concept of tamoxifen treatment in patients with advanced disease may also be resurrected, and its therapeutic efficacy tested against biomarkers such as *KRAS* status and hormone receptor expression.

## Supplementary information

**Additional file 1: Data 1.** Specification of the targeted gene panel, showing types of mutations and tumor morphology in each case for every 70 genes sequenced.

**Additional file 2: Table S1.** Intercorrelation between ER and PR expression in the entire cohort and stratified by sex and morphology.

**Additional file 3: Table S2.** Associations of ER expression status (negative vs positive) with patient and tumor characteristics in the entire cohort, intestinal-type tumors and pancreatobiliary-type tumors, allover and stratified by sex.

**Additional file 4: Table S3.** Associations of PR expression status (negative vs positive) with patient and tumor characteristics in the entire cohort, intestinal-type tumors and pancreatobiliary-type tumors, allover and stratified by sex.

**Additional file 5: Table S4.** Associations of ER expression status (negative vs positive) with common mutations in the entire cohort, intestinal-type tumors and pancreatobiliary-type tumors, allover and stratified by sex.

**Additional file 6: Table S5.** Associations of PR expression status (negative vs positive) with common mutations in the entire cohort, intestinal-type tumors and pancreatobiliary-type tumors, allover and stratified by sex.

## Data Availability

Part of the data generated and analyzed during this study are included in this published article. A data sheet of the analyzed genes (*n* = 70), including the NGS data, is provided in Additional file 1: Data 1 as well as in the article by Lundgren et al. [40]. The raw data of hormone receptor expression will be made available upon request. Patient and clinicopathological data of this cohort are not publicly available due to its content of identifiable human data. Requests to access the datasets should be directed to Dr. Gustav Andersson.

## Abbreviations

CI: Confidence interval
ER: Estrogen receptor-α
FFPE: Formalin-fixed paraffin-embedded
HR: Hazard ratio
I-type: Intestinal type
MCN: Mucinous cystic neoplasm
OS: Overall survival
OTS: Ovarian-type stroma
PB-type: Pancreatobiliary type
PR: Progesterone receptor
RFS: Recurrence-free survival
TCGA: The Cancer Genome Atlas
TMA: Tissue microarray
