# Supplementary material for: Clinical significance of stromal ER and PR expression in periampullary adenocarcinoma
Source: Biomark Res. 2019 Nov 19;7:26. doi: 10.1186/s40364-019-0176-9 (PMC6862740; doi:10.1186/s40364-019-0176-9)
Supplement: Supplementary file 2 — Additional file 2: Table S1. Intercorrelation between ER and PR expression in the entire cohort and stratified by sex and morphology. [file 40364_2019_176_MOESM2_ESM.docx]

**Table S1.** Intercorrelation between ER and PR expression in the entire cohort and stratified by sex and morphology.

|  | **All** | **Women** | **Men** |
| --- | --- | --- | --- |
| **All** |  |  |  |
| *R* | 0.37** | 0.28* | 0.48** |
| p | < 0.001 | 0.011 | < 0.001 |
| n | 162 | 80 | 82 |
| **I-type** |  |  |  |
| *R* | 0.22 | 0.20 | 0.25 |
| p | 0.105 | 0.286 | 0.227 |
| n | 58 | 32 | 26 |
| **PB-type** |  |  |  |
| *R* | 0.42** | 0.30* | 0.54** |
| p | < 0.001 | 0.036 | < 0.001 |
| n | 104 | 48 | 56 |

All correlation coefficients are Spearman Rho.
*Correlation is significant at the 0.05 level (two-tailed);
**Correlation is significant at the 0.01 level (two-tailed);
I-type = Intestinal-type;
PB-type = Pancreatobiliary-type
