# Supplementary material for: Clinical significance of stromal ER and PR expression in periampullary adenocarcinoma
Source: Biomark Res. 2019 Nov 19;7:26. doi: 10.1186/s40364-019-0176-9 (PMC6862740; doi:10.1186/s40364-019-0176-9)
Supplement: Supplementary file 3 — Additional file 3: Table S2. Associations of ER expression status (negative vs positive) with patient and tumor characteristics in the entire cohort, intestinal-type tumors and pancreatobiliary-type tumors, allover and stratified by sex. [file 40364_2019_176_MOESM3_ESM.docx]

**Table S2.** Associations of ER expression status (negative vs positive) with patient and tumor characteristics in the entire cohort, intestinal-type tumors and pancreatobiliary-type tumors, allover and stratified by sex.

| **Entire cohort** | | | | | | | | | |
| --- | --- | --- | --- | --- | --- | --- | --- | --- | --- |
|  | **All** | | | **Women** | | | **Men** | | |
|  | **ER- (n=120)** | **ER+ (n=49)** | *P* | **ER- (n=49)** | **ER+ (n=35)** | *P* | **ER- (n=71)** | **ER+ (n=14)** | *P* |
| **Age (years)** |  |  |  |  |  |  |  |  |  |
| Mean | 65.1 | 66.4 | *0.315* | 63.8 | 68.0 | *0.034* | 66.0 | 62.3 | *0.158* |
| Median | 66.5 | 67.0 |  | 65.0 | 70.0 |  | 67.0 | 63.5 |  |
| S.D. | 8.8 | 8.9 |  | 9.9 | 8.5 |  | 7.9 | 8.8 |  |
| Range | 38.0 - 83.0 | 44.0 - 81.0 |  | 38.0 - 81.0 | 48.0 - 81.0 |  | 43.0 - 83.0 | 44.0 - 74.0 |  |
| **Sex** |  |  |  |  |  |  |  |  |  |
| Women | 49 | 35 | *<0.001* | - | - |  | - | - |  |
| Men | 71 | 14 |  | - | - |  | - | - |  |
| **Tumor origin** |  |  |  |  |  |  |  |  |  |
| Duodenum | 10 | 4 | *0.210* | 3 | 3 | *0.300* | 7 | 1 | *0.290* |
| Ampulla intestinal type | 37 | 12 |  | 19 | 9 |  | 18 | 3 |  |
| Ampulla pancreatobiliary type | 16 | 3 |  | 6 | 3 |  | 10 | 0 |  |
| Distal bile duct | 30 | 15 |  | 11 | 10 |  | 19 | 5 |  |
| Pancreas | 27 | 15 |  | 10 | 10 |  | 17 | 5 |  |
| **Tumor size (mm)** |  |  |  |  |  |  |  |  |  |
| Mean | 29.5 | 29.6 | *0.718* | 28.1 | 29.4 | *0.525* | 30.5 | 30.1 | *0.957* |
| Median | 30.0 | 30.0 |  | 30.0 | 30.0 |  | 30.0 | 30.0 |  |
| S.D. | 13.9 | 9.9 |  | 13.0 | 9.3 |  | 14.5 | 11.5 |  |
| Range | 5.0 - 90.0 | 10.0 - 60.0 |  | 5.0 - 70.0 | 10.0 - 50.0 |  | 5.0 - 90.0 | 15.0 - 60.0 |  |
| **Differentiation grade** |  |  |  |  |  |  |  |  |  |
| Well - Moderate | 55 | 15 | *0.069* | 18 | 10 | *0.437* | 37 | 5 | *0.265* |
| Poor - Undifferentiated | 65 | 34 |  | 31 | 25 |  | 34 | 9 |  |
| **T-stage** |  |  |  |  |  |  |  |  |  |
| T1 | 4 | 2 | *0.055* | 1 | 0 | *0.660* | 3 | 2 | *0.013* |
| T2 | 13 | 8 |  | 7 | 5 |  | 6 | 3 |  |
| T3 | 69 | 33 |  | 29 | 25 |  | 40 | 8 |  |
| T4 | 34 | 6 |  | 12 | 5 |  | 22 | 1 |  |
| **N-stage** |  |  |  |  |  |  |  |  |  |
| N0 | 42 | 21 | *0.120* | 20 | 17 | *0.248* | 22 | 4 | *0.754* |
| N1 | 43 | 20 |  | 16 | 13 |  | 27 | 7 |  |
| N2 | 35 | 8 |  | 13 | 5 |  | 22 | 3 |  |
| **Resection margin** |  |  |  |  |  |  |  |  |  |
| R0 | 13 | 10 | *0.101* | 6 | 7 | *0.336* | 7 | 3 | *0.222* |
| R1 - Rx | 107 | 39 |  | 43 | 28 |  | 64 | 11 |  |
| **Perineural growth** |  |  |  |  |  |  |  |  |  |
| No | 47 | 19 | *0.962* | 22 | 15 | *0.854* | 25 | 4 | *0.634* |
| Yes | 73 | 30 |  | 27 | 20 |  | 46 | 10 |  |
| **Invasion of lymphatic vessels** |  |  |  |  |  |  |  |  |  |
| No | 42 | 18 | *0.831* | 20 | 14 | *0.940* | 22 | 4 | *0.859* |
| Yes | 78 | 31 |  | 29 | 21 |  | 49 | 10 |  |
| **Invasion of blood vessels** |  |  |  |  |  |  |  |  |  |
| No | 90 | 37 | *0.945* | 36 | 27 | *0.703* | 54 | 10 | *0.715* |
| Yes | 30 | 12 |  | 13 | 8 |  | 17 | 4 |  |
| **Growth in peripancreatic fat** |  |  |  |  |  |  |  |  |  |
| No | 44 | 19 | *0.798* | 24 | 16 | *0.769* | 20 | 3 | *0.606* |
| Yes | 76 | 30 |  | 25 | 19 |  | 51 | 11 |  |
| **Adjuvant chemotherapy** |  |  |  |  |  |  |  |  |  |
| None | 72 | 23 | *0.187* | 32 | 17 | *0.176* | 40 | 6 | *0.297* |
| 5-FU analogue | 9 | 4 |  | 4 | 4 |  | 5 | 0 |  |
| Gemcitabine | 32 | 18 |  | 11 | 11 |  | 21 | 7 |  |
| Gemcitabine + Capecitabine | 1 | 2 |  | 0 | 2 |  | 1 | 0 |  |
| Oxaliplatin + 5-FU analogue | 4 | 1 |  | 2 | 1 |  | 2 | 0 |  |
| Gemcitabine + Oxaliplatin | 2 | 1 |  | 0 | 0 |  | 2 | 1 |  |
| **Intestinal-type** | | | | | | | | | |
|  | **All** | | | **Women** | | | **Men** | | |
|  | **ER- (n=47)** | **ER+ (n=16)** | *P* | **ER- (n=22)** | **ER+ (n=12)** | *P* | **ER- (n=25)** | **ER+ (n=4)** | *P* |
| **Age (years)** |  |  |  |  |  |  |  |  |  |
| Mean | 64.4 | 64.8 | *0.887* | 62.2 | 66.9 | *0.136* | 66.2 | 58.5 | *0.082* |
| Median | 67.0 | 66.5 |  | 64.5 | 69.0 |  | 67.0 | 61.5 |  |
| S.D. | 10.0 | 9.2 |  | 9.8 | 8.4 |  | 10.0 | 9.5 |  |
| Range | 38.0 - 83.0 | 45.0 - 77.0 |  | 38.0 - 78.0 | 48.0 - 77.0 |  | 43.0 - 83.0 | 45.0 - 66.0 |  |
| **Sex** |  |  |  |  |  |  |  |  |  |
| Women | 22 | 12 | *0.053* | - | - | *-* | - | - | *-* |
| Men | 25 | 4 |  | - | - |  | - | - |  |
| **Tumor origin** |  |  |  |  |  |  |  |  |  |
| Duodenum | 10 | 4 | *0.759* | 3 | 3 | *0.413* | 7 | 1 | *0.903* |
| Ampulla intestinal type | 37 | 12 |  | 19 | 9 |  | 18 | 3 |  |
| Ampulla pancreatobiliary type | *-* | *-* |  | - | - |  | - | - |  |
| Distal bile duct | *-* | *-* |  | - | - |  | - | - |  |
| Pancreas | *-* | *-* |  | - | - |  | - | - |  |
| **Tumor size (mm)** |  |  |  |  |  |  |  |  |  |
| Mean | 28.0 | 30.4 | *0.471* | 27.9 | 30.2 | *0.709* | 28.1 | 31.3 | *0.784* |
| Median | 26.0 | 28.5 |  | 30.0 | 28.5 |  | 25.0 | 25.0 |  |
| S.D. | 16.5 | 13.8 |  | 15.0 | 12.2 |  | 18.0 | 20.2 |  |
| Range | 5.0 - 90.0 | 10.0 - 60.0 |  | 5.0 - 50.0 | 10.0 - 50.0 |  | 5.0 - 90.0 | 15.0 - 60.0 |  |
| **Differentiation grade** |  |  |  |  |  |  |  |  |  |
| Well - Moderate | 24 | 7 | *0.616* | 9 | 4 | *0.669* | 15 | 3 | *0.573* |
| Poor - Undifferentiated | 23 | 9 |  | 13 | 8 |  | 10 | 1 |  |
| **T-stage** |  |  |  |  |  |  |  |  |  |
| T1 | 3 | 1 | *0.743* | 1 | 0 | *0.550* | 2 | 1 | *0.235* |
| T2 | 9 | 2 |  | 5 | 1 |  | 4 | 1 |  |
| T3 | 16 | 9 |  | 9 | 8 |  | 7 | 1 |  |
| T4 | 19 | 4 |  | 7 | 3 |  | 12 | 1 |  |
| **N-stage** |  |  |  |  |  |  |  |  |  |
| N0 | 23 | 10 | *0.362* | 13 | 9 | *0.632* | 10 | 1 | *0.826* |
| N1 | 15 | 4 |  | 6 | 1 |  | 9 | 3 |  |
| N2 | 9 | 2 |  | 3 | 2 |  | 6 | 0 |  |
| **Resection margin** |  |  |  |  |  |  |  |  |  |
| R0 | 11 | 6 | *0.276* | 5 | 5 | *0.254* | 6 | 1 | *0.966* |
| R1 - Rx | 36 | 10 |  | 17 | 7 |  | 19 | 3 |  |
| **Perineural growth** |  |  |  |  |  |  |  |  |  |
| No | 31 | 13 | *0.253* | 16 | 10 | *0.492* | 15 | 3 | *0.573* |
| Yes | 16 | 3 |  | 6 | 2 |  | 10 | 1 |  |
| **Invasion of lymphatic vessels** |  |  |  |  |  |  |  |  |  |
| No | 21 | 8 | *0.715* | 10 | 6 | *0.803* | 11 | 2 | *0.826* |
| Yes | 26 | 8 |  | 12 | 6 |  | 14 | 2 |  |
| **Invasion of blood vessels** |  |  |  |  |  |  |  |  |  |
| No | 43 | 15 | *0.774* | 19 | 11 | *0.651* | 24 | 4 | *0.689* |
| Yes | 4 | 1 |  | 3 | 1 |  | 1 | 0 |  |
| **Growth in peripancreatic fat** |  |  |  |  |  |  |  |  |  |
| No | 29 | 12 | *0.339* | 16 | 9 | *0.888* | 13 | 3 | *0.399* |
| Yes | 18 | 4 |  | 6 | 3 |  | 12 | 1 |  |
| **Adjuvant chemotherapy** |  |  |  |  |  |  |  |  |  |
| None | 33 | 12 | *0.986* | 16 | 8 | *0.482* | 17 | 4 | *0.274* |
| 5-FU analogue | 5 | 0 |  | 3 | 0 |  | 2 | 0 |  |
| Gemcitabine | 5 | 2 |  | 1 | 2 |  | 4 | 0 |  |
| Gemcitabine + Capecitabine | 0 | 1 |  | 0 | 1 |  | 0 | 0 |  |
| Oxaliplatin + 5-FU analogue | 3 | 1 |  | 2 | 1 |  | 1 | 0 |  |
| Gemcitabine + Oxaliplatin | 1 | 0 |  | 0 | 0 |  | 1 | 0 |  |
| **Pancreatobiliary-type** | | | | | | | | | |
|  | **All** | | | **Women** | | | **Men** | | |
|  | **ER- (n=73)** | **ER+ (n=33)** | *P* | **ER- (n=27)** | **ER+ (n=23)** | *P* | **ER- (n=46)** | **ER+ (n=10)** | *P* |
| **Age (years)** |  |  |  |  |  |  |  |  |  |
| Mean | 65.6 | 67.1 | *0.267* | 65.1 | 68.5 | *0.185* | 65.9 | 63.8 | *0.645* |
| Median | 66.0 | 68.0 |  | 65.0 | 70.0 |  | 67.0 | 64.5 |  |
| S.D. | 7.9 | 8.7 |  | 9.9 | 8.6 |  | 6.7 | 8.6 |  |
| Range | 44.0 - 81.0 | 44.0 - 81.0 |  | 44.0 - 81.0 | 48.0 - 81.0 |  | 48.0 - 78.0 | 44.0 - 74.0 |  |
| **Sex** |  |  |  |  |  |  |  |  |  |
| Women | 27 | 23 | *0.002* | - | - | *-* | - | - | *-* |
| Men | 46 | 10 |  | - | - |  | - | - |  |
| **Tumor origin** |  |  |  |  |  |  |  |  |  |
| Duodenum | *-* | *-* |  | - | - |  | - | - |  |
| Ampulla intestinal type | *-* | *-* |  | - | - |  | - | - |  |
| Ampulla pancreatobiliary type | 16 | 3 | *0.165* | 6 | 3 | *0.455* | 10 | 0 | *0.173* |
| Distal bile duct | 30 | 15 |  | 11 | 10 |  | 19 | 5 |  |
| Pancreas | 27 | 15 |  | 10 | 10 |  | 17 | 5 |  |
| **Tumor size (mm)** |  |  |  |  |  |  |  |  |  |
| Mean | 30.5 | 29.2 | *0.904* | 28.3 | 29.0 | *0.568* | 31.8 | 29.7 | *0.812* |
| Median | 30.0 | 30.0 |  | 26.0 | 30.0 |  | 30.0 | 30.0 |  |
| S.D. | 11.8 | 7.5 |  | 11.3 | 7.7 |  | 12.1 | 7.5 |  |
| Range | 5.0 - 70.0 | 15.0 - 40.0 |  | 9.0 - 70.0 | 15.0 - 40.0 |  | 5.0 - 70.0 | 15.0 - 40.0 |  |
| **Differentiation grade** |  |  |  |  |  |  |  |  |  |
| Well - Moderate | 31 | 8 | *0.073* | 9 | 6 | *0.581* | 22 | 2 | *0.110* |
| Poor - Undifferentiated | 42 | 25 |  | 18 | 17 |  | 24 | 8 |  |
| **T-stage** |  |  |  |  |  |  |  |  |  |
| T1 | 1 | 1 | *0.012* | 0 | 0 |  | 1 | 1 | *0.016* |
| T2 | 4 | 6 |  | 2 | 4 | *0.175* | 2 | 2 |  |
| T3 | 53 | 24 |  | 20 | 17 |  | 33 | 7 |  |
| T4 | 15 | 2 |  | 5 | 2 |  | 10 | 0 |  |
| **N-stage** |  |  |  |  |  |  |  |  |  |
| N0 | 19 | 11 | *0.125* | 7 | 8 | *0.125* | 12 | 3 | *0.750* |
| N1 | 28 | 16 |  | 10 | 12 |  | 18 | 4 |  |
| N2 | 26 | 6 |  | 10 | 3 |  | 16 | 3 |  |
| **Resection margin** |  |  |  |  |  |  |  |  |  |
| R0 | 2 | 4 | *0.054* | 1 | 2 | *0.463* | 1 | 2 | *0.025* |
| R1 - Rx | 71 | 29 |  | 26 | 21 |  | 45 | 8 |  |
| **Perineural growth** |  |  |  |  |  |  |  |  |  |
| No | 16 | 6 | *0.662* | 6 | 5 | *0.968* | 10 | 1 | *0.401* |
| Yes | 57 | 27 |  | 21 | 18 |  | 36 | 9 |  |
| **Invasion of lymphatic vessels** |  |  |  |  |  |  |  |  |  |
| No | 21 | 10 | *0.873* | 10 | 8 | *0.870* | 11 | 2 | *0.792* |
| Yes | 52 | 23 |  | 17 | 15 |  | 35 | 8 |  |
| **Invasion of blood vessels** |  |  |  |  |  |  |  |  |  |
| No | 47 | 22 | *0.820* | 17 | 16 | *0.627* | 30 | 6 | *0.757* |
| Yes | 26 | 11 |  | 10 | 7 |  | 16 | 4 |  |
| **Growth in peripancreatic fat** |  |  |  |  |  |  |  |  |  |
| No | 15 | 7 | *0.938* | 8 | 7 | *0.951* | 7 | 0 | *0.191* |
| Yes | 58 | 26 |  | 19 | 16 |  | 39 | 10 |  |
| **Adjuvant chemotherapy** |  |  |  |  |  |  |  |  |  |
| None | 39 | 11 | *0.124* | 16 | 9 | *0.269* | 23 | 2 | *0.064* |
| 5-FU analogue | 4 | 4 |  | 1 | 4 |  | 3 | 0 |  |
| Gemcitabine | 27 | 16 |  | 10 | 9 |  | 17 | 7 |  |
| Gemcitabine + Capecitabine | 1 | 1 |  | 0 | 1 |  | 1 | 0 |  |
| Oxaliplatin + 5-FU analogue | 1 | 0 |  | 0 | 0 |  | 1 | 0 |  |
| Gemcitabine + Oxaliplatin | 1 | 1 |  | 0 | 0 |  | 1 | 1 |  |
