# Supplementary material for: Clinical significance of stromal ER and PR expression in periampullary adenocarcinoma
Source: Biomark Res. 2019 Nov 19;7:26. doi: 10.1186/s40364-019-0176-9 (PMC6862740; doi:10.1186/s40364-019-0176-9)
Supplement: Supplementary file 4 — Additional file 4: Table S3. Associations of PR expression status (negative vs positive) with patient and tumor characteristics in the entire cohort, intestinal-type tumors and pancreatobiliary-type tumors, allover and stratified by sex. [file 40364_2019_176_MOESM4_ESM.docx]

**Table S3.** Associations of PR expression status (negative vs positive) with patient and tumor characteristics in the entire cohort, intestinal-type tumors and pancreatobiliary-type tumors, allover and stratified by sex.

| **Entire cohort** | | | | | | | | | |
| --- | --- | --- | --- | --- | --- | --- | --- | --- | --- |
|  | **All** | | | **Women** | | | **Men** | | |
|  | **PR- (n=113)** | **PR+ (n=50)** | *P* | **PR- (n=52)** | **PR+ (n=28)** | *P* | **PR- (n=61)** | **PR+ (n=22)** | *P* |
| **Age (years)** |  |  |  |  |  |  |  |  |  |
| Mean | 66.3 | 66.1 | *0.944* | 65.9 | 67.0 | *0.499* | 66.7 | 64.9 | *0.383* |
| Median | 67.0 | 66.5 |  | 67.0 | 68.5 |  | 67.0 | 66.0 |  |
| S.D. | 7.8 | 8.1 |  | 8.4 | 8.9 |  | 7.2 | 7.0 |  |
| Range | 44.0 - 83.0 | 44.0 - 81.0 |  | 44.0 - 81.0 | 48.0 - 81.0 |  | 48.0 - 83.0 | 44.0 - 74.0 |  |
| **Sex** |  |  |  |  |  |  |  |  |  |
| Women | 52 | 28 | *0.241* | - | - | *-* | - | - | *-* |
| Men | 61 | 22 |  | - | - |  | - | - |  |
| **Tumor origin** |  |  |  |  |  |  |  |  |  |
| Duodenum | 13 | 1 | *0.003* | 5 | 1 | *0.018* | 8 | 0 | *0.055* |
| Ampulla intestinal type | 35 | 9 |  | 21 | 5 |  | 14 | 4 |  |
| Ampulla pancreatobiliary type | 14 | 5 |  | 6 | 3 |  | 8 | 2 |  |
| Distal bile duct | 26 | 19 |  | 10 | 11 |  | 16 | 8 |  |
| Pancreas | 25 | 16 |  | 10 | 8 |  | 15 | 8 |  |
| **Tumor size (mm)** |  |  |  |  |  |  |  |  |  |
| Mean | 30.0 | 29.2 | *0.677* | 29.2 | 28.8 | *0.931* | 30.6 | 29.9 | *0.656* |
| Median | 30.0 | 26.0 |  | 30.0 | 27.5 |  | 30.0 | 26.0 |  |
| S.D. | 13.2 | 12.2 |  | 11.6 | 11.5 |  | 14.5 | 13.4 |  |
| Range | 5.0 - 90.0 | 9.0 - 70.0 |  | 5.0 - 70.0 | 9.0 - 50.0 |  | 5.0 - 90.0 | 15.0 - 70.0 |  |
| **Differentiation grade** |  |  |  |  |  |  |  |  |  |
| Well - Moderate | 48 | 18 | *0.439* | 18 | 8 | *0.584* | 30 | 10 | *0.766* |
| Poor - Undifferentiated | 65 | 32 |  | 34 | 20 |  | 31 | 12 |  |
| **T-stage** |  |  |  |  |  |  |  |  |  |
| T1 | 4 | 2 | *0.139* | 1 | 0 | *0.158* | 3 | 2 | *0.420* |
| T2 | 12 | 7 |  | 5 | 5 |  | 7 | 2 |  |
| T3 | 66 | 34 |  | 32 | 20 |  | 34 | 14 |  |
| T4 | 31 | 7 |  | 14 | 3 |  | 17 | 4 |  |
| **N-stage** |  |  |  |  |  |  |  |  |  |
| N0 | 44 | 16 | *0.857* | 27 | 8 | *0.321* | 17 | 8 | *0.577* |
| N1 | 39 | 23 |  | 13 | 15 |  | 26 | 8 |  |
| N2 | 30 | 11 |  | 12 | 5 |  | 18 | 6 |  |
| **Resection margin** |  |  |  |  |  |  |  |  |  |
| R0 | 16 | 5 | *0.466* | 8 | 3 | *0.565* | 8 | 2 | *0.621* |
| R1 - Rx | 97 | 45 |  | 44 | 25 |  | 53 | 20 |  |
| **Perineural growth** |  |  |  |  |  |  |  |  |  |
| No | 50 | 13 | *0.028* | 26 | 9 | *0.127* | 24 | 4 | *0.074* |
| Yes | 63 | 37 |  | 26 | 19 |  | 37 | 18 |  |
| **Invasion of lymphatic vessels** |  |  |  |  |  |  |  |  |  |
| No | 45 | 12 | *0.051* | 24 | 7 | *0.066* | 21 | 5 | *0.313* |
| Yes | 68 | 38 |  | 28 | 21 |  | 40 | 17 |  |
| **Invasion of blood vessels** |  |  |  |  |  |  |  |  |  |
| No | 89 | 33 | *0.084* | 40 | 20 | *0.591* | 49 | 13 | *0.051* |
| Yes | 24 | 17 |  | 12 | 8 |  | 12 | 9 |  |
| **Growth in peripancreatic fat** |  |  |  |  |  |  |  |  |  |
| No | 46 | 14 | *0.122* | 26 | 12 | *0.544* | 20 | 2 | *0.032* |
| Yes | 67 | 36 |  | 26 | 16 |  | 41 | 20 |  |
| **Adjuvant chemotherapy** |  |  |  |  |  |  |  |  |  |
| None | 66 | 26 | *0.429* | 32 | 16 | *0.677* | 34 | 10 | *0.378* |
| 5-FU analogue | 8 | 4 |  | 5 | 3 |  | 3 | 1 |  |
| Gemcitabine | 33 | 16 |  | 13 | 7 |  | 20 | 9 |  |
| Gemcitabine + Capecitabine | 1 | 1 |  | 0 | 1 |  | 1 | 0 |  |
| Oxaliplatin + 5-FU analogue | 3 | 2 |  | 2 | 1 |  | 1 | 1 |  |
| Gemcitabine + Oxaliplatin | 2 | 1 |  | 0 | 0 |  | 2 | 1 |  |
| **Intestinal-type** | | | | | | | | | |
|  | **All** | | | **Women** | | | **Men** | | |
|  | **PR- (n=48)** | **PR+ (n=10)** | *P* | **PR- (n=26)** | **PR+ (n=6)** | *P* | **PR- (n=22)** | **PR+ (n=4)** | *P* |
| **Age (years)** |  |  |  |  |  |  |  |  |  |
| Mean | 65.9 | 67.9 | *0.433* | 64.6 | 67.7 | *0.436* | 67.5 | 68.3 | *1.000* |
| Median | 67.0 | 69.0 |  | 66.0 | 69.0 |  | 67.0 | 69.0 |  |
| S.D. | 8.2 | 6.2 |  | 8.1 | 7.5 |  | 8.1 | 4.5 |  |
| Range | 44.0 - 83.0 | 56.0 - 76.0 |  | 44-0 - 78.0 | 56.0 - 76.0 |  | 48.0 - 83.0 | 63.0 - 72.0 |  |
| **Sex** |  |  |  |  |  |  |  |  |  |
| Women | 26 | 6 | *0.738* | - | - | *-* | - | - | *-* |
| Men | 22 | 4 |  | - | - |  | - | - |  |
| **Tumor origin** |  |  |  |  |  |  |  |  |  |
| Duodenum | 13 | 1 | *0.255* | 5 | 1 | *0.886* | 8 | 0 | *0.155* |
| Ampulla intestinal type | 35 | 9 |  | 21 | 5 |  | 14 | 4 |  |
| Ampulla pancreatobiliary type | *-* | *-* |  | - | - |  | - | - |  |
| Distal bile duct | *-* | *-* |  | - | - |  | - | - |  |
| Pancreas | *-* | *-* |  | - | - |  | - | - |  |
| **Tumor size (mm)** |  |  |  |  |  |  |  |  |  |
| Mean | 29.8 | 25.6 | *0.556* | 29.4 | 30.2 | *0.760* | 30.3 | 18.8 | *0.389* |
| Median | 30.0 | 20.0 |  | 30.0 | 30.5 |  | 26.5 | 17.5 |  |
| S.D. | 16.6 | 14.3 |  | 13.3 | 17.2 |  | 20.2 | 4.8 |  |
| Range | 5.0 - 90.0 | 10.0 - 50.0 |  | 5.0 - 50.0 | 10.0 - 50.0 |  | 5.0 - 90.0 | 15.0 - 25.0 |  |
| **Differentiation grade** |  |  |  |  |  |  |  |  |  |
| Well - Moderate | 22 | 6 | *0.419* | 9 | 3 | *0.490* | 13 | 3 | *0.555* |
| Poor - Undifferentiated | 26 | 4 |  | 17 | 3 |  | 9 | 1 |  |
| **T-stage** |  |  |  |  |  |  |  |  |  |
| T1 | 3 | 1 | *0.177* | 1 | 0 | *0.135* | 2 | 1 | *0.608* |
| T2 | 6 | 4 |  | 2 | 3 |  | 4 | 1 |  |
| T3 | 21 | 2 |  | 14 | 2 |  | 7 | 0 |  |
| T4 | 18 | 3 |  | 9 | 1 |  | 9 | 2 |  |
| **N-stage** |  |  |  |  |  |  |  |  |  |
| N0 | 25 | 5 | *0.479* | 17 | 3 | *0.911* | 8 | 2 | *0.372* |
| N1 | 13 | 5 |  | 4 | 3 |  | 9 | 2 |  |
| N2 | 10 | 0 |  | 5 | 0 |  | 5 | 0 |  |
| **Resection margin** |  |  |  |  |  |  |  |  |  |
| R0 | 12 | 3 | *0.745* | 6 | 2 | *0.607* | 6 | 1 | *0.926* |
| R1 - Rx | 36 | 7 |  | 20 | 4 |  | 16 | 3 |  |
| **Perineural growth** |  |  |  |  |  |  |  |  |  |
| No | 34 | 7 | *0.958* | 19 | 5 | *0.607* | 15 | 2 | *0.491* |
| Yes | 14 | 3 |  | 7 | 1 |  | 7 | 2 |  |
| **Invasion of lymphatic vessels** |  |  |  |  |  |  |  |  |  |
| No | 21 | 6 | *0.353* | 12 | 3 | *0.867* | 9 | 3 | *0.217* |
| Yes | 27 | 4 |  | 14 | 3 |  | 13 | 1 |  |
| **Invasion of blood vessels** |  |  |  |  |  |  |  |  |  |
| No | 44 | 9 | *0.866* | 23 | 5 | *0.736* | 21 | 4 | *0.670* |
| Yes | 4 | 1 |  | 3 | 1 |  | 1 | 0 |  |
| **Growth in peripancreatic fat** |  |  |  |  |  |  |  |  |  |
| No | 32 | 6 | *0.689* | 19 | 4 | *0.757* | 13 | 2 | *0.740* |
| Yes | 16 | 4 |  | 7 | 2 |  | 9 | 2 |  |
| **Adjuvant chemotherapy** |  |  |  |  |  |  |  |  |  |
| None | 35 | 7 | *0.249* | 18 | 5 | *0.982* | 17 | 2 | *0.088* |
| 5-FU analogue | 4 | 0 |  | 3 | 0 |  | 1 | 0 |  |
| Gemcitabine | 6 | 1 |  | 3 | 0 |  | 3 | 1 |  |
| Gemcitabine + Capecitabine | 0 | 0 |  | 0 | 0 |  | 0 | 0 |  |
| Oxaliplatin + 5-FU analogue | 3 | 1 |  | 2 | 1 |  | 1 | 0 |  |
| Gemcitabine + Oxaliplatin | 0 | 1 |  | 0 | 0 |  | 0 | 1 |  |
| **Pancreatobiliary-type** | | | | | | | | | |
|  | **All** | | | **Women** | | | **Men** | | |
|  | **PR- (n=65)** | **PR+ (n=40)** | *P* | **PR- (n=26)** | **PR+ (n=22)** | *P* | **PR- (n=39)** | **PR+ (n=18)** | *P* |
| **Age (years)** |  |  |  |  |  |  |  |  |  |
| Mean | 66.6 | 65.6 | *0.637* | 67.1 | 66.9 | *0.992* | 66.3 | 64.1 | *0.335* |
| Median | 67.0 | 66.0 |  | 68.5 | 67.5 |  | 67.0 | 64.5 |  |
| S.D. | 7.5 | 8.5 |  | 8.7 | 9.4 |  | 6.8 | 7.3 |  |
| Range | 48.0 - 81.0 | 44.0 - 81.0 |  | 51.0 - 81.0 | 48.0 - 81.0 |  | 48.0 - 78.0 | 44.0 - 74.0 |  |
| **Sex** |  |  |  |  |  |  |  |  |  |
| Women | 26 | 22 | *0.136* | - | - | *-* | - | - | *-* |
| Men | 39 | 18 |  | - | - |  | - | - |  |
| **Tumor origin** |  |  |  |  |  |  |  |  |  |
| Duodenum | - | - |  | - | - |  | - | - |  |
| Ampulla intestinal type | - | - |  | - | - |  | - | - |  |
| Ampulla pancreatobiliary type | 14 | 5 | *0.471* | 6 | 3 | *0.730* | 8 | 2 | *0.461* |
| Distal bile duct | 26 | 19 |  | 10 | 11 |  | 16 | 8 |  |
| Pancreas | 25 | 16 |  | 10 | 8 |  | 15 | 8 |  |
| **Tumor size (mm)** |  |  |  |  |  |  |  |  |  |
| Mean | 30.1 | 30.2 | *0.829* | 29.0 | 28.4 | *0.992* | 30.8 | 32.3 | *0.945* |
| Median | 30.0 | 30.0 |  | 30.0 | 27.5 |  | 30.0 | 30.0 |  |
| S.D. | 10.2 | 11.7 |  | 10.0 | 10.0 |  | 10.3 | 13.5 |  |
| Range | 5.0 - 70.0 | 9.0 - 70.0 |  | 15.0 - 70.0 | 9.0 - 45.0 |  | 5.0 - 55.0 | 15.0 - 70.0 |  |
| **Differentiation grade** |  |  |  |  |  |  |  |  |  |
| Well - Moderate | 26 | 12 | *0.303* | 9 | 5 | *0.372* | 17 | 7 | *0.741* |
| Poor - Undifferentiated | 39 | 28 |  | 17 | 17 |  | 22 | 11 |  |
| **T-stage** |  |  |  |  |  |  |  |  |  |
| T1 | 1 | 1 | *0.374* | 0 | 0 | *0.598* | 1 | 1 | *0.458* |
| T2 | 6 | 3 |  | 3 | 2 |  | 3 | 1 |  |
| T3 | 45 | 32 |  | 18 | 18 |  | 27 | 14 |  |
| T4 | 13 | 4 |  | 5 | 2 |  | 8 | 2 |  |
| **N-stage** |  |  |  |  |  |  |  |  |  |
| N0 | 19 | 11 | *0.920* | 10 | 5 | *0.598* | 9 | 6 | *0.643* |
| N1 | 26 | 18 |  | 9 | 12 |  | 17 | 6 |  |
| N2 | 20 | 11 |  | 7 | 5 |  | 13 | 6 |  |
| **Resection margin** |  |  |  |  |  |  |  |  |  |
| R0 | 4 | 2 | *0.806* | 2 | 1 | *0.657* | 2 | 1 | *0.947* |
| R1 - Rx | 61 | 38 |  | 24 | 21 |  | 37 | 17 |  |
| **Perineural growth** |  |  |  |  |  |  |  |  |  |
| No | 16 | 6 | *0.242* | 7 | 4 | *0.477* | 9 | 2 | *0.292* |
| Yes | 49 | 34 |  | 19 | 18 |  | 30 | 16 |  |
| **Invasion of lymphatic vessels** |  |  |  |  |  |  |  |  |  |
| No | 24 | 6 | *0.016* | 12 | 4 | *0.043* | 12 | 2 | *0.112* |
| Yes | 41 | 34 |  | 14 | 18 |  | 27 | 16 |  |
| **Invasion of blood vessels** |  |  |  |  |  |  |  |  |  |
| No | 45 | 24 | *0.336* | 17 | 15 | *0.839* | 28 | 9 | *0.112* |
| Yes | 20 | 16 |  | 9 | 7 |  | 11 | 9 |  |
| **Growth in peripancreatic fat** |  |  |  |  |  |  |  |  |  |
| No | 14 | 8 | *0.851* | 7 | 8 | *0.487* | 7 | 0 | *0.057* |
| Yes | 51 | 32 |  | 19 | 14 |  | 32 | 18 |  |
| **Adjuvant chemotherapy** |  |  |  |  |  |  |  |  |  |
| None | 31 | 19 | *0.773* | 14 | 11 | *0.825* | 17 | 8 | *0.806* |
| 5-FU analogue | 4 | 4 |  | 2 | 3 |  | 2 | 1 |  |
| Gemcitabine | 27 | 15 |  | 10 | 7 |  | 17 | 8 |  |
| Gemcitabine + Capecitabine | 1 | 1 |  | 0 | 1 |  | 1 | 0 |  |
| Oxaliplatin + 5-FU analogue | 0 | 1 |  | 0 | 0 |  | 0 | 1 |  |
| Gemcitabine + Oxaliplatin | 2 | 0 |  | 0 | 0 |  | 2 | 0 |  |
