# Supplementary material for: Clinical significance of stromal ER and PR expression in periampullary adenocarcinoma
Source: Biomark Res. 2019 Nov 19;7:26. doi: 10.1186/s40364-019-0176-9 (PMC6862740; doi:10.1186/s40364-019-0176-9)
Supplement: Supplementary file 5 — Additional file 5: Table S4. Associations of ER expression status (negative vs positive) with common mutations in the entire cohort, intestinal-type tumors and pancreatobiliary-type tumors, allover and stratified by sex. [file 40364_2019_176_MOESM5_ESM.docx]

**Table S4.** Associations of ER expression status (negative vs positive) with common mutations in the entire cohort, intestinal-type tumors and pancreatobiliary-type tumors, allover and stratified by sex.

| **Entire cohort** | | | | | | | | | |
| --- | --- | --- | --- | --- | --- | --- | --- | --- | --- |
|  | **All** | | | **Women** | | | **Men** | | |
|  | **ER- (n=75)** | **ER+ (n=25)** | *P* | **ER- (n=31)** | **ER+ (n=16)** | *P* | **ER- (n=44)** | **ER+ (n=9)** | *P* |
| **APC** |  |  |  |  |  |  |  |  |  |
| Wild-type | 69 | 20 | *0.097* | 26 | 12 | *0.464* | 43 | 8 | *0.205* |
| Mutated | 6 | 5 |  | 5 | 4 |  | 1 | 1 |  |
| **CDK2NA** |  |  |  |  |  |  |  |  |  |
| Wild-type | 64 | 23 | *0.391* | 25 | 14 | *0.553* | 39 | 9 | *0.288* |
| Mutated | 11 | 2 |  | 6 | 2 |  | 5 | 0 |  |
| **ERBB3** |  |  |  |  |  |  |  |  |  |
| Wild-type | 66 | 23 | *0.580* | 27 | 14 | *0.969* | 39 | 9 | *0.288* |
| Mutated | 9 | 2 |  | 4 | 2 |  | 5 | 0 |  |
| **KRAS** |  |  |  |  |  |  |  |  |  |
| Wild-type | 41 | 13 | *0.817* | 17 | 8 | *0.753* | 24 | 5 | *0.956* |
| Mutated | 34 | 12 |  | 14 | 8 |  | 20 | 4 |  |
| **NF1** |  |  |  |  |  |  |  |  |  |
| Wild-type | 68 | 22 | *0.700* | 27 | 13 | *0.594* | 41 | 9 | *0.420* |
| Mutated | 7 | 3 |  | 4 | 3 |  | 3 | 0 |  |
| **RNF43** |  |  |  |  |  |  |  |  |  |
| Wild-type | 66 | 21 | *0.607* | 26 | 13 | *0.821* | 40 | 8 | *0.850* |
| Mutated | 9 | 4 |  | 5 | 3 |  | 4 | 1 |  |
| **SMAD4** |  |  |  |  |  |  |  |  |  |
| Wild-type | 67 | 22 | *0.854* | 29 | 11 | *0.024* | 38 | 9 | *0.239* |
| Mutated | 8 | 3 |  | 2 | 5 |  | 6 | 0 |  |
| **SMARCA4** |  |  |  |  |  |  |  |  |  |
| Wild-type | 39 | 11 | *0.488* | 26 | 14 | *0.741* | 41 | 8 | *0.657* |
| Mutated | 36 | 14 |  | 5 | 2 |  | 3 | 1 |  |
| **TP53** |  |  |  |  |  |  |  |  |  |
| Wild-type | 36 | 10 | *0.489* | 19 | 7 | *0.252* | 20 | 4 | *0.956* |
| Mutated | 39 | 15 |  | 12 | 9 |  | 24 | 5 |  |
| **Intestinal-type** | | | | | | | | | |
|  | **All** | | | **Women** | | | **Men** | | |
|  | **ER- (n=31)** | **ER+ (n=9)** | *P* | **ER- (n=14)** | **ER+ (n=6)** | *P* | **ER- (n=17)** | **ER+ (n=3)** | *P* |
| **APC** |  |  |  |  |  |  |  |  |  |
| Wild-type | 25 | 4 | *0.032* | 9 | 2 | *0.202* | 16 | 2 | *0.144* |
| Mutated | 6 | 5 |  | 5 | 4 |  | 1 | 1 |  |
| **CDK2NA** |  |  |  |  |  |  |  |  |  |
| Wild-type | 30 | 9 | *0.585* | 14 | 6 | *-* | 16 | 3 | *0.666* |
| Mutated | 1 | 0 |  | 0 | 0 |  | 1 | 0 |  |
| **ERBB3** |  |  |  |  |  |  |  |  |  |
| Wild-type | 25 | 7 | *0.850* | 11 | 4 | *0.573* | 14 | 3 | *0.430* |
| Mutated | 6 | 2 |  | 3 | 2 |  | 3 | 0 |  |
| **KRAS** |  |  |  |  |  |  |  |  |  |
| Wild-type | 18 | 5 | *0.893* | 7 | 3 | *1.000* | 11 | 2 | *0.948* |
| Mutated | 13 | 4 |  | 7 | 3 |  | 6 | 1 |  |
| **NF1** |  |  |  |  |  |  |  |  |  |
| Wild-type | 28 | 8 | *0.900* | 13 | 5 | *0.515* | 15 | 3 | *0.531* |
| Mutated | 3 | 1 |  | 1 | 1 |  | 2 | 0 |  |
| **RNF43** |  |  |  |  |  |  |  |  |  |
| Wild-type | 27 | 7 | *0.491* | 13 | 5 | *0.515* | 14 | 2 | *0.531* |
| Mutated | 4 | 2 |  | 1 | 1 |  | 3 | 1 |  |
| **SMAD4** |  |  |  |  |  |  |  |  |  |
| Wild-type | 28 | 7 | *0.316* | 13 | 4 | *0.133* | 15 | 3 | *0.531* |
| Mutated | 3 | 2 |  | 1 | 2 |  | 2 | 0 |  |
| **SMARCA4** |  |  |  |  |  |  |  |  |  |
| Wild-type | 28 | 8 | *0.900* | 13 | 5 | *0.515* | 15 | 3 | *0.531* |
| Mutated | 3 | 1 |  | 1 | 1 |  | 2 | 0 |  |
| **TP53** |  |  |  |  |  |  |  |  |  |
| Wild-type | 19 | 4 | *0.368* | 10 | 2 | *0.111* | 9 | 2 | *0.660* |
| Mutated | 12 | 5 |  | 4 | 4 |  | 8 | 1 |  |
| **Pancreatobiliary-type** | | | | | | | | | |
|  | **All** | | | **Women** | | | **Men** | | |
|  | **ER- (n=44)** | **ER+ (n=16)** | *P* | **ER- (n=17)** | **ER+ (n=10)** | *P* | **ER- (n=27)** | **ER+ (n=6)** | *P* |
| **APC** |  |  |  |  |  |  |  |  |  |
| Wild-type | 44 | 16 | *-* | 17 | 10 | *-* | 27 | 6 | *-* |
| Mutated | 0 | 0 |  | 0 | 0 |  | 0 | 0 |  |
| **CDK2NA** |  |  |  |  |  |  |  |  |  |
| Wild-type | 34 | 14 | *0.381* | 11 | 8 | *0.401* | 23 | 6 | *0.315* |
| Mutated | 10 | 2 |  | 6 | 2 |  | 4 | 0 |  |
| **ERBB3** |  |  |  |  |  |  |  |  |  |
| Wild-type | 41 | 16 | *0.284* | 16 | 10 | *0.434* | 25 | 6 | *0.492* |
| Mutated | 3 | 0 |  | 1 | 0 |  | 2 | 0 |  |
| **KRAS** |  |  |  |  |  |  |  |  |  |
| Wild-type | 23 | 8 | *0.876* | 10 | 5 | *0.656* | 13 | 3 | *0.935* |
| Mutated | 21 | 8 |  | 7 | 5 |  | 14 | 3 |  |
| **NF1** |  |  |  |  |  |  |  |  |  |
| Wild-type | 40 | 14 | *0.697* | 14 | 8 | *0.879* | 26 | 6 | *0.632* |
| Mutated | 4 | 2 |  | 3 | 2 |  | 1 | 0 |  |
| **RNF43** |  |  |  |  |  |  |  |  |  |
| Wild-type | 39 | 14 | *0.903* | 13 | 8 | *0.831* | 26 | 6 | *0.632* |
| Mutated | 5 | 2 |  | 4 | 2 |  | 1 | 0 |  |
| **SMAD4** |  |  |  |  |  |  |  |  |  |
| Wild-type | 39 | 13 | *0.457* | 16 | 7 | *0.088* | 23 | 6 | *0.315* |
| Mutated | 5 | 3 |  | 1 | 3 |  | 4 | 0 |  |
| **SMARCA4** |  |  |  |  |  |  |  |  |  |
| Wild-type | 39 | 14 | *0.903* | 13 | 9 | *0.382* | 26 | 5 | *0.229* |
| Mutated | 5 | 2 |  | 4 | 1 |  | 1 | 1 |  |
| **TP53** |  |  |  |  |  |  |  |  |  |
| Wild-type | 20 | 7 | *0.907* | 9 | 5 | *0.883* | 11 | 2 | *0.737* |
| Mutated | 24 | 9 |  | 8 | 5 |  | 16 | 4 |  |
