# Supplementary material for: Clinical significance of stromal ER and PR expression in periampullary adenocarcinoma
Source: Biomark Res. 2019 Nov 19;7:26. doi: 10.1186/s40364-019-0176-9 (PMC6862740; doi:10.1186/s40364-019-0176-9)
Supplement: Supplementary file 6 — Additional file 6: Table S5. Associations of PR expression status (negative vs positive) with common mutations in the entire cohort, intestinal-type tumors and pancreatobiliary-type tumors, allover and stratified by sex. [file 40364_2019_176_MOESM6_ESM.docx]

**Table S5.** Associations of PR expression status (negative vs positive) with common mutations in the entire cohort, intestinal-type tumors and pancreatobiliary-type tumors, allover and stratified by sex.

| **Entire cohort** | | | | | | | | | |
| --- | --- | --- | --- | --- | --- | --- | --- | --- | --- |
|  | **All** | | | **Women** | | | **Men** | | |
|  | **PR- (n=68)** | **PR+ (n=29)** | *P* | **PR- (n=30)** | **PR+ (n=15)** | *P* | **PR- (n=38)** | **PR+ (n=14)** | *P* |
| **APC** |  |  |  |  |  |  |  |  |  |
| Wild-type | 60 | 26 | *0.840* | 24 | 12 | *1.000* | 36 | 14 | *0.381* |
| Mutated | 8 | 3 |  | 6 | 3 |  | 2 | 0 |  |
| **CDK2NA** |  |  |  |  |  |  |  |  |  |
| Wild-type | 60 | 24 | *0.469* | 25 | 12 | *0.783* | 35 | 12 | *0.488* |
| Mutated | 8 | 5 |  | 5 | 3 |  | 3 | 2 |  |
| **ERBB3** |  |  |  |  |  |  |  |  |  |
| Wild-type | 57 | 29 | *0.021* | 24 | 15 | *0.063* | 33 | 14 | *0.153* |
| Mutated | 11 | 0 |  | 6 | 0 |  | 5 | 0 |  |
| **KRAS** |  |  |  |  |  |  |  |  |  |
| Wild-type | 39 | 12 | *0.149* | 18 | 6 | *0.205* | 21 | 6 | *0.427* |
| Mutated | 29 | 17 |  | 12 | 9 |  | 17 | 8 |  |
| **NF1** |  |  |  |  |  |  |  |  |  |
| Wild-type | 61 | 26 | *0.994* | 26 | 13 | *1.000* | 35 | 13 | *0.928* |
| Mutated | 7 | 3 |  | 4 | 2 |  | 3 | 1 |  |
| **RNF43** |  |  |  |  |  |  |  |  |  |
| Wild-type | 58 | 26 | *0.564* | 24 | 13 | *0.581* | 34 | 13 | *0.714* |
| Mutated | 10 | 3 |  | 6 | 2 |  | 4 | 1 |  |
| **SMAD4** |  |  |  |  |  |  |  |  |  |
| Wild-type | 60 | 24 | *0.469* | 26 | 12 | *0.561* | 34 | 12 | *0.707* |
| Mutated | 8 | 5 |  | 4 | 3 |  | 4 | 2 |  |
| **SMARCA4** |  |  |  |  |  |  |  |  |  |
| Wild-type | 60 | 26 | *0.840* | 26 | 13 | *1.000* | 34 | 13 | *0.714* |
| Mutated | 8 | 3 |  | 4 | 2 |  | 4 | 1 |  |
| **TP53** |  |  |  |  |  |  |  |  |  |
| Wild-type | 34 | 14 | *0.876* | 14 | 11 | *0.090* | 20 | 3 | *0.044* |
| Mutated | 34 | 15 |  | 16 | 4 |  | 18 | 11 |  |
| **Intestinal-type** | | | | | | | | | |
|  | **All** | | | **Women** | | | **Men** | | |
|  | **PR- (n=30)** | **PR+ (n=6)** | *P* | **PR- (n=14)** | **PR+ (n=4)** | *P* | **PR- (n=16)** | **PR+ (n=2)** | *P* |
| **APC** |  |  |  |  |  |  |  |  |  |
| Wild-type | 22 | 3 | *0.257* | 8 | 1 | *0.257* | 14 | 2 | *0.596* |
| Mutated | 8 | 3 |  | 6 | 3 |  | 2 | 0 |  |
| **CDK2NA** |  |  |  |  |  |  |  |  |  |
| Wild-type | 29 | 6 | *0.650* | 14 | 4 | *-* | 15 | 2 | *0.716* |
| Mutated | 1 | 0 |  | 0 | 0 |  | 1 | 0 |  |
| **ERBB3** |  |  |  |  |  |  |  |  |  |
| Wild-type | 22 | 6 | *0.151* | 9 | 4 | *0.160* | 13 | 2 | *0.502* |
| Mutated | 8 | 0 |  | 5 | 0 |  | 3 | 0 |  |
| **KRAS** |  |  |  |  |  |  |  |  |  |
| Wild-type | 18 | 2 | *0.230* | 8 | 1 | *0.257* | 10 | 1 | *0.732* |
| Mutated | 12 | 4 |  | 6 | 3 |  | 6 | 1 |  |
| **NF1** |  |  |  |  |  |  |  |  |  |
| Wild-type | 28 | 5 | *0.418* | 14 | 3 | *0.054* | 14 | 2 | *0.596* |
| Mutated | 2 | 1 |  | 0 | 1 |  | 2 | 0 |  |
| **RNF43** |  |  |  |  |  |  |  |  |  |
| Wild-type | 26 | 4 | *0.230* | 13 | 3 | *0.316* | 13 | 1 | *0.316* |
| Mutated | 4 | 2 |  | 1 | 1 |  | 3 | 1 |  |
| **SMAD4** |  |  |  |  |  |  |  |  |  |
| Wild-type | 28 | 3 | *0.005* | 13 | 2 | *0.043* | 15 | 1 | *0.063* |
| Mutated | 2 | 3 |  | 1 | 2 |  | 1 | 1 |  |
| **SMARCA4** |  |  |  |  |  |  |  |  |  |
| Wild-type | 27 | 6 | *0.418* | 13 | 4 | *0.582* | 14 | 2 | *0.596* |
| Mutated | 3 | 0 |  | 1 | 0 |  | 2 | 0 |  |
| **TP53** |  |  |  |  |  |  |  |  |  |
| Wild-type | 17 | 4 | *0.650* | 7 | 4 | *0.070* | 10 | 0 | *0.094* |
| Mutated | 13 | 2 |  | 7 | 0 |  | 6 | 2 |  |
| **Pancreatobiliary-type** | | | | | | | | | |
|  | **All** | | | **Women** | | | **Men** | | |
|  | **PR- (n=38)** | **PR+ (n=23)** | *P* | **PR- (n=16)** | **PR+ (n=11)** | *P* | **PR- (n=22)** | **PR+ (n=12)** | *P* |
| **APC** |  |  |  |  |  |  |  |  |  |
| Wild-type | 38 | 23 | *-* | 16 | 11 | *-* | 22 | 12 | *-* |
| Mutated | 0 | 0 |  | 0 | 0 |  | 0 | 0 |  |
| **CDK2NA** |  |  |  |  |  |  |  |  |  |
| Wild-type | 31 | 18 | *0.752* | 11 | 8 | *0.824* | 20 | 10 | *0.512* |
| Mutated | 7 | 5 |  | 5 | 3 |  | 2 | 2 |  |
| **ERBB3** |  |  |  |  |  |  |  |  |  |
| Wild-type | 35 | 23 | *0.167* | 15 | 11 | *0.398* | 20 | 12 | *0.282* |
| Mutated | 3 | 0 |  | 1 | 0 |  | 2 | 0 |  |
| **KRAS** |  |  |  |  |  |  |  |  |  |
| Wild-type | 21 | 10 | *0.372* | 10 | 5 | *0.381* | 11 | 5 | *0.642* |
| Mutated | 17 | 13 |  | 6 | 6 |  | 11 | 7 |  |
| **NF1** |  |  |  |  |  |  |  |  |  |
| Wild-type | 33 | 21 | *0.596* | 12 | 10 | *0.296* | 21 | 11 | *0.654* |
| Mutated | 5 | 2 |  | 4 | 1 |  | 1 | 1 |  |
| **RNF43** |  |  |  |  |  |  |  |  |  |
| Wild-type | 32 | 22 | *0.174* | 11 | 10 | *0.174* | 21 | 12 | *0.453* |
| Mutated | 6 | 1 |  | 5 | 1 |  | 1 | 0 |  |
| **SMAD4** |  |  |  |  |  |  |  |  |  |
| Wild-type | 32 | 21 | *0.426* | 13 | 10 | *0.488* | 19 | 11 | *0.646* |
| Mutated | 6 | 2 |  | 3 | 1 |  | 3 | 1 |  |
| **SMARCA4** |  |  |  |  |  |  |  |  |  |
| Wild-type | 33 | 20 | *0.990* | 13 | 9 | *0.970* | 20 | 11 | *0.941* |
| Mutated | 5 | 3 |  | 3 | 2 |  | 2 | 1 |  |
| **TP53** |  |  |  |  |  |  |  |  |  |
| Wild-type | 17 | 10 | *0.924* | 7 | 7 | *0.310* | 10 | 3 | *0.241* |
| Mutated | 21 | 13 |  | 9 | 4 |  | 12 | 9 |  |
